# Telehealth Postpandemic: A Model for Michigan and Beyond

**DOI:** 10.1089/tmj.2024.0519

**Published:** 2024-12-06

**Authors:** Bree E. Holtz, Charles R. Doarn

**Affiliations:** ^1^ Department of Advertising and Public Relations, College of Communication Arts & Sciences, Michigan State University, East Lansing, Michigan, USA.; ^2^ Department of Environmental and Public Health Sciences, College of Medicine, University of Cincinnati, Cincinnati, Ohio, USA.

**Keywords:** telehealth, telemedicine, pandemic, strategy, equity

## Abstract

During the COVID-19 pandemic, telemedicine and telehealth saw a groundswell of growth, only to be shackled in the aftermath of the Public Health Emergency. A Think Tank, funded by Agency for Healthcare Research and Quality, was held at Michigan State University in August 2024. This paper serves as an introduction to a series of articles focusing on the evolution and future of telehealth in a postpandemic world. It highlights key themes including patient equity, technology, clinical opportunities, research, and education, using Michigan as a model for national adaptation. The paper aims to ignite further discussion and innovation within the telehealth community.

## Introduction

While telemedicine and telehealth have been around for the past more than 60 years,^1^ it has been limited in scope and widespread adoption. Telehealth, for decades, has been limited to a few specialties (i.e., imaging, psychiatry, prison populations, etc.), research demonstration projects, or a spattering of clinics with providers who offered it. However, during the beginning months of the COVID-19 pandemic, telehealth proliferated seemingly overnight.^2^ In fact, the pandemic accelerated the adoption of telehealth, thrusting it to the forefront of health care delivery.

Initially, many health care systems had envisioned a gradual integration of telehealth over several years, yet the pandemic necessitated its immediate implementation. In fact, the Centers for Disease Control and Prevention reported a 71.1% increase from 2019 to 2021.^3^ This in many cases was precipitated by the relaxation of guidelines, policies, and laws.^2,4^ Alongside the accelerated growth in clinical use, there has been a substantial rise in academic research focused on the implementation, adoption, and use of telehealth services. A quick PubMed search illustrates this surge: between 1971 and 2019 (49 years), 74,083 academic journal articles on telemedicine and telehealth were published. Remarkably, from 2020 to July 2024 alone, 42,460 articles have been added, reflecting a significant surge in research activity in this field.^5^

This swift transition, while crucial, left little time for systematic evaluation of telehealth’s deployment and efficacy.^6–8^ As we settle into the “new normal,” it is imperative to rethink and refine the strategies surrounding telehealth to ensure its sustainability and effectiveness. Therefore, a collaborative effort involving providers, patient advocates, health systems, insurers, policymakers, technology providers, and researchers is needed to advance the practice of telehealth.

## Methods

In response to these challenges, the Agency for Healthcare Research and Quality (AHRQ) funded a Telehealth Think Tank titled “Telehealth Postpandemic: A Roadmap for Michigan.” This initiative convened a diverse group of stakeholders, including telemedicine and telehealth experts, health care providers, patient advocates, government staffers, and insurers, to address the critical question: “What’s next?”

Michigan is an ideal setting for this conference because of its racial, political, cultural, economic, and geographic diversity. This diversity provides a unique opportunity to develop telehealth strategies that cater to a broad range of needs, including those of rural, urban, Indigenous, and migrant populations.^9^ Concentrating on one state allowed us to have a focused discussion with clear implications and recommendations that can then be disseminated and tailored to other states. We believe the outcomes of this work will allow other states to tailor practices that suit their needs.

This Think Tank brought together 30 individuals with a range of expertise in this space, including health system representatives, physicians, researchers, patient advocates, insurance, government, technology facilitators, and medical educators. Together, this group developed perspectives and recommendations for telehealth in the future. This carefully selected group gathered for one full day and two half-days in East Lansing, MI, where they engaged in dynamic discussions and explored critical questions. The event cultivated an atmosphere of thoughtful introspection and intense discourse. As a result of these exchanges, the participants developed a collection of white papers that addressed the central themes shaping the future of telehealth.

## Key Themes and Applications

This event provided a collaborative platform for stakeholders to share insights and develop actionable strategies. The conference format, which included keynote addresses and workgroup sessions, facilitated the cross-pollination of ideas and fostered potential collaborations. The insights gained from these discussions can inform the development of telehealth solutions that are effective, scalable, and sustainable not only in Michigan but also nationally. Their discussions, captured in a series of papers, outline critical areas for innovation, including technology, patient equity, clinical practice, education, and research.

Several papers discuss telehealth’s application to improve care in underserved areas, with maternal health emerging as a notable example. Two papers, in particular, highlight how telehealth, including Remote Patient Monitoring (RPM), can address health care gaps in maternal care deserts and improve access to postpartum services. While maternal care is just one example, these discussions illustrate the broader capacity of telehealth to target specific populations facing health care inequities.^10–11^

Telehealth’s transformative potential is closely tied to advancements in RPM. Several papers highlight how wearable devices and artificial intelligence-driven technologies enable real-time patient monitoring, allowing providers to intervene earlier and enhance patient outcomes. However, for these benefits to be fully realized, significant investments in digital infrastructure—including broadband expansion and improved digital literacy—are essential. These technological advances, combined with community engagement and education, are critical to ensuring that telehealth becomes a sustainable and equitable component of health care delivery.^10–11^

The theme of value-based care also emerges as central to telehealth’s evolution. One paper discusses how telehealth can be integrated into care models to improve patient outcomes and enhance health care efficiency and financial sustainability. However, this requires more than just technological upgrades; it demands substantial investments in provider education and patient engagement to ensure that telehealth does not exacerbate existing inequities. Several papers emphasize the importance of community engagement and comprehensive education to overcome barriers such as digital literacy and access to technology—particularly in rural and underserved areas. These efforts are vital for ensuring that telehealth reaches its full potential, offering equitable access to all patients, regardless of their circumstances.^10–12^

Mentioned in several of the papers, education plays a pivotal role in telehealth’s success. The BEACH model for shared decision-making, introduced in one of the papers, offers a structured framework for engaging patients in their care by fostering rapport, educating them about their options, and supporting patient autonomy. This approach is crucial as telehealth becomes more integrated into everyday health care, ensuring that patients remain active participants in their care.^12–13^

Finally, the research roadmap highlights the importance of continued interdisciplinary research into telehealth’s long-term impact on clinical outcomes, patient engagement, technology integration, and ethical considerations. Building a robust body of evidence will be essential for guiding policymakers and practitioners in making telehealth an equitable, effective tool for health care delivery.^14^

## Future Directions

As we look ahead, it is clear that telehealth has the potential to be a transformative force in health care, but it requires sustained momentum. The papers from this Think Tank provide a comprehensive blueprint for moving telehealth forward. A call to action is clear: telehealth must be solidified as a permanent part of the healthcare system, with investments in infrastructure, education, and policy reforms that make telehealth more accessible and equitable. The lessons learned from the pandemic must not be forgotten; instead, they should fuel innovation, ensuring that telehealth continues to evolve and meet the diverse needs of all populations.

By integrating advancements in technology, prioritizing education, and addressing healthcare inequities, telehealth can truly become a lasting and impactful solution. The path forward involves collaboration across disciplines and sectors to ensure that telehealth is not only sustained but expanded to provide high-quality, equitable care for all.

## Conclusions

This Think Tank laid a robust foundation for future telehealth developments. By leveraging Michigan’s unique demographic and economic diversity, the conference served as a model for the sustainable and scalable implementation of telehealth practices. Ongoing evaluation and dissemination are crucial to maintaining momentum and ensuring that telehealth continues evolving and meeting diverse populations’ needs.^2^ We encourage you to share these papers with your colleagues, as well as with state and federal representatives, to help ensure that telehealth remains accessible and sustainable for everyone. This paper captures the essence of the conversations and invites further contributions from the telehealth community to continue driving innovation and addressing the complex challenges of modern health care. By fostering collaboration and dialogue among stakeholders, we can ensure that telehealth remains at the forefront of health care innovation and continues to provide equitable and effective care for all.

## Disclosure Statement

No competing financial interests exist.
